# Molecular characterization of nontuberculous Mycobacteria in a tuberculosis and HIV reference unit in the State of Amazonas, Brazil

**DOI:** 10.1590/0037-8682-0613-2021

**Published:** 2022-08-05

**Authors:** Ana Carolina de Oliveira de Lima, Karen Barros Schmid, Hilda Ferreira de Melo, Rafaella Christine Athayde, Rossiclea Lins Monte, Isabela Neves de Almeida, Silvana Spíndola de Miranda, Afrânio Kritski, Maria Lucia Rossetti, Marcelo Cordeiros-Santos

**Affiliations:** 1 Fundação Oswaldo Cruz, Instituto Oswaldo Cruz, Rio de Janeiro, RJ, Brasil.; 2 Universidade do Estado do Amazonas, Programa de Pós-Graduação em Medicina Tropical, Manaus, AM, Brasil.; 3 Secretaria de Estado da Saúde do Rio Grande do Sul, Centro de Desenvolvimento Científico e Tecnológico, Porto Alegre, RS, Brasil.; 4 Fundação de Medicina Tropical Dr. Heitor Vieira Dourado, Manaus, AM, Brasil.; 5 Universidade Federal de Ouro Preto, Escola de Farmácia, Departamento de Análises Clínicas, Ouro Preto, MG, Brasil.; 6 Universidade Federal de Minas Gerais, Faculdade de Medicina, Departamento de Clínica Médica/Pneumologia/Tisologia, Belo Horizonte, MG, Brasil.; 7 Universidade Federal do Rio de Janeiro, Escola de Medicina, Programa Acadêmico de Tuberculose, Rio de Janeiro, RJ, Brasil.; 8 Universidade Luterana do Brasil, Programa de Pós-Graduação em Biologia Molecular e Celular, Canoas, RS, Brasil.

**Keywords:** HIV infections, Molecular diagnostic technique, Nontuberculous mycobacteria, Prevalence

## Abstract

**Background:**

In recent years, the prevalence of nontuberculous mycobacterial (NTM) infections has increased in different regions of the world. The American Thoracic Society (ATS) recommends standardized identification criteria, reinforcing the need for faster and less complicated clinical and laboratory techniques.

**Methods:**

In this retrospective study, NTM species isolated from pulmonary, extrapulmonary, and disseminated samples from patients treated at a TB/HIV reference unit in the State of Amazonas from 2011 to 2014 were identified through a combination of molecular techniques.

**Results:**

To identify the molecular technique, 50 cryopreserved NTM cultures were recovered and subcultivated in culture medium. The potentially pathogenic NTM species identified were *M. avium*, *M. intracellulare*, *M. kansasii*, *M. chelonae*, *M. abscessus*, *M. fortuitum,* and *M. peregrinum*. Results of GenoType^®^ showed moderate agreement with those of genomic sequencing (kappa = 0.60), whereas the results obtained by the PRA-hsp65 technique disagreed with the results obtained by sequencing (kappa = 0.49).

**Conclusions:**

Our findings highlight that GenoType CM is a good method for the identification of NTM, as well as the need for the application of standardized criteria, such as those set forth by the ATS.

## INTRODUCTION

In recent years, the prevalence of infections caused by nontuberculous mycobacteria (NTM) has increased in different regions of the world ^1^ ^,^ ^2^ .

One of the most common occupations in the northern region of Brazil is fishing, and recent studies have highlighted fishermen and other individuals exposed to fish as a population with a greater risk of developing skin infections caused by *Mycobacerium marinum* ^3^ ^,^ ^4^ . In Manaus, an outbreak of postoperative NTM infection was related to the water supplied to the surgical center ^5^ .

Furthermore, people infected with HIV are more prone to diseases caused by NTM ^6^ . Thus, surveillance of these infections is of utmost importance, and correct diagnosis to differentiate between cases of colonization and disease requires well-defined clinical, radiological, and laboratory criteria.

The diagnosis of diseases caused by NTM is still a major challenge due to the non-specific clinical symptoms, possible transitory colonization, or contamination. In addition to the possibility of infection, whether associated or not, to mycobacteria of the *Mycobacterium tuberculosis* complex, which all present similar signs and symptoms ^7^ ^-^ ^9^ .

Recently updated guidelines for the treatment of pulmonary disease caused by NTM, developed in conjunction with the American Thoracic Society (ATS), European Respiratory Society (ERS), European Society of Clinical Microbiology and Infectious Diseases (ESCMID), and Infectious Diseases Society of America (IDSA), suggest that more than one isolated culture (≥2) of NTM, revealing the same species of NTM or isolated subspecies ^10^ , is necessary to confirm the disease. 

In addition, it is recommended that isolates be subjected to drug sensitivity tests (DST). In species of the *Mycobacterium avium* complex (MAC), mutations in the 23S rRNA gene confer resistance to macrolides and mutations in the 16S rRNA gene confer resistance to amikacin and/or related aminoglycosides. Species such as *M. kansasii* and *M. abscessus* are resistant to macrolides and amikacin, with rifampicin and clarithromycin being the principal drugs tested for the *M. kansasii* species ^11^ . These findings reinforce the need for swifter and less cumbersome techniques to identify NTM species in clinical and laboratory routines to guarantee adequate medical treatment.

In this context, the present study aimed to identify isolated NTMs in patients who received medical care in a TB/HIV reference unit in the state of Amazonas, using a combination of molecular techniques.

## METHODS

### Study design

This retrospective cohort study was conducted from 2011 to 2014 at the Fundação de Medicina Tropical Dr. Heitor Vieira Dourado (FMT-HVD), a TB/HIV reference center in the city of Manaus, Amazonas state, in the northern region of Brazil. 

This study was carried out using two methods: 1) Analysis of the clinical and sociodemographic data of the patients, and 2) Molecular characterization of NMT.

### Data analysis

Clinical and laboratory data of the patients were searched using the online medical records system, I-Doctor, made available at FMT-HVD, and the Notifiable Diseases Information System (SINAN, in Portuguese). Data analysis was conducted using a databank constructed with the Microsoft Excel computer program (Office 2016), in which the information contained in the clinical and laboratory records (sex, age, HIV, viral load, lymphocyte count, antiretroviral therapy used (ART), biological sample, result of the mycobacterial culture, and prescribed medicines) was recorded. Biological samples were classified as pulmonary, extrapulmonary, or disseminated. Phlegm and gastric juices were classified as pulmonary in origin, while cerebrospinal fluid, urine, tracheal, and abscess secretions, and skin and organ biopsies were classified as extrapulmonary in origin. Blood and bone marrow samples were classified as having a disseminated origin.

For the molecular characterization of NTM, patients whose clinical samples presented positive culture results for NTM and negative culture results for *M. tuberculosis Complex* (MTC) were included. 

The separation of MTC was conducted through microscopic and macroscopic analyses of the culture and growth inhibition in a Löwenstein-Jensen (Becton Dickinson^©^) culture medium containing p-nitrobenzoic acid (LJ-PNB) and niacin (Becton Dickinson^©^) ( Supplementary Material 1).

### Extraction of the genomic DNA

To conduct the molecular tests, this study used DNA from the NTM cultures that were cryopreserved at -70ºC and subcultivated in Ogawa Kudoh (OK - Laborclin^©^) solid medium. Subcultivation of the cryopreserved strains was performed to verify the viability of the strain and to identify possible contaminants ^12^ .

Cryopreserved NTM cultures were recovered and subcultivated in culture medium. 

In order to exclude MTC, the macroscopic characteristics of the colonies were considered, such as the presence of growth and/or the presence of contamination in the culture medium, the morphology and pigmentation of the colonies, and the microscopic features of the colony stained by the Ziehl-Neelsen method. The formation of the cord and the presence of contamination by other bacteria and fungi were also evaluated. In the analysis of the subcultivations, 10.7% (6/56) presented contaminants and the absence of mycobacterial growth, and were not included in the molecular tests.

After this screening step, the genomic DNA of *Mycobacteria* was extracted using the cetyltrimethylammonium bromide (CTAB; SIGMA-Aldrich-Merck^©^) method. It was performed briefly after bacterial inactivation by heating for 30 min at 80 °C, followed by the enzymatic reaction by applying lysozyme (SIGMA-Aldrich-Merck^©^) (10 mg/mL) and proteinase K (SIGMA-Aldrich-Merck^©^) (10 mg/mL) solution. The DNA was purified by adding CTAB and was precipitated by using alcohol solutions of chloroform/isoamyl alcohol (24:1), isopropanol, and 70% ethanol ^13^ .

### GenoType^®^ Mycobacterium CM-AS - Genotype

The GenoType^®^ Mycobacterium CM-AS Mobius Life Science^©^ (Genotype) trial was conducted according to the manufacturer’s instructions. The complete procedure was divided into three steps: (i) DNA extraction from the cultivated material through sonication, (ii) Multiplex amplification with biotinylated primers, and (iii) Reverse hybridization ^14^ .

*Molecular identification by Polymerase Chain Reaction Restriction Analysis of the hsp65 gene- PRA-hsp65* **(** Supplementary Material 2 **)**. 

Amplification and digestion of the *hsp65* gene were performed using the *BstE*II and *Hae*III restriction enzymes. The resulting fragments were subjected to electrophoretic analysis using agarose gel, and the determination of the species was carried out by the interpretation of the bands in the gel, according to the profile of the DNA fragments obtained through digestion by each specific enzyme ^15^ .

### Genome Sequencing - hsp65 Gene

For *hsp65* gene sequencing, the following primer were used: Hsp667 F 5’ GGCCAAGACAATTGCGTACG; Hsp667 R 5’ GGAGCTGACCAGCAGGATG. The conditions were as follows: 2 min at 95 °C, followed by 30 cycles for 45 s, 57 °C for 45 s, and 72 °C for 45 s, with a final extension at 72 °C for 5 min, generating a fragment of 439 bp. Sequencing was performed using the same oligonucleotides ^16^ .

### Gene rpoB

Sequencing of the *rpoB* gene was performed using the following primers: Myco F 5’ GCAAGGTCACCCCGAAGGG and Myco R 5’ AGCGGCTGCTGGGTGATCATC, under the following amplification conditions: 95 °C for 1 min, followed by 35 cycles of denaturation at 94 °C for 30 s, 64 °C for 30 s, 72°C for 90 s, and one final extension of 72°C for 5 min, generating a fragment of 764 bp. The same oligonucleotides were used for the sequencing of the amplicons ^16^ .

The amplicons were first purified with polyethylene glycol (PEG) 8000/2.5 M NaCl using the ABI 3500 Genetic Analyzer with capillaries of 50 cm and a POP7 polymer (Applied Biosystems^®^), and then again purified using the BigDye XTerminator Purification Kit (Applied Biosystems^®^) ^17^ . Sequencing was used as the standard method for NTM characterization.

### Sequence analysis

The electrostatic profiles were determined and compared to the restriction patterns of various algorithms found in the *PRASITE* online system (http://app.chuv.ch/prasite/index.html)

The sequencing reaction products were aligned and analyzed using the DNASTAR Lasergene7 SeqMan program and compared using the Basic Local Alignment Search Tool (BLAST) databank ^17^ .

The sequences were analyzed with higher coverage, with an average coverage of 98.8%. For species identification, a similarity value equal to or higher than 97% with the reference sequences was considered. 

### Statistical analyses

Statistical analysis was conducted using the SPSS v.21 statistical program (SPSS Ins. Chicago, IL, USA). The agreement between the methods was evaluated using the kappa score. 

### Ethics

This study was approved by the FMT-HVD Research Ethics Committee and logged under the protocol number CAAE: 58214916.8.0000.0005. 

## RESULTS

During the study period, 3202 patients with a likely infection caused by mycobacteria, including presumed TB cases, received medical care at FMT-HVD. Of these, 758 (23.7%) presented a positive culture for mycobacteria, among which 224 (29.5%) were identified as NTM.

Of the 224 positive cultures for NTM, 189 (84.3%) were of pulmonary origin, 20 (8.9%) from extrapulmonary, and 15 (6.6%) from disseminated origin. According to sample source, 187 (83.5%) of the positive samples were phlegm samples and 2 (0.9%) were gastric juice samples, followed by tracheal and abscess secretions at 11 (4.9%), skin and organ biopsies at 6 (2.7%), urine at 2 (0.9%), cerebrospinal fluid at 1 (0.4%), blood at 13 (5.8%), and bone marrow at 2 (0.9%).

### Analysis of Clinical Characteristics of patients with NTM

Among the 224 patients with NTM, 66% were men, with a median age of 35 years. HIV serology results were obtained for 87 (38.8%) patients, of whom 84 (37.5%) presented an HIV-positive serology and 3 (1.3%) presented an HIV-negative serology. The sociodemographic, clinical, and laboratory data are described in Table 1 **,** and the NTM species identified according to the anatomic site and HIV serology results are described in Table 2.

**TABLE 1:** Main clinical and laboratory characteristics of patients with isolates of NonTuberculous Mycobacteria.

	N	Percentage/IQR
Positive HIV Serology	84/224	37.5%
Negative HIV Serology	3/224	1.3%
Lymphocyte Count CD4 ≥100	29/84	34.5%
ART use	16/84	19.1%
No information of ART use	68/84	80.9%
	**Median**	**IQR**
Viral charge	17490.5	1.899-63.3360
Lymphocyte Count CD4	244	74.5-383.5

**Legend: IQR:** Interquartile Range; **ART:** Anti-retroviral therapy; **HIV:** Human Immunodeficiency Virus; **N:** number of patients.

**TABLE 2:** Species distribution by anatomical site of identified Nontuberculous Mycobacteria and Human Immunodeficiency Virus serology (N=50).

	Pulmonary	Extrapulmonary/Disseminated					
Identification ^*a*^	(n = 44)	(n = 6)			HIV		
Species	Sputum	Blood	Bone Marrow	Secretion	HIV+ (n=38)	HIV- (n=3)	NA (n=9)
*M. gordonae*	15 (30%)	-	-	-	10 (20%)	1 (2%)	4 (8%)
*Mycobaterium* sp*.*	10 (20%)	-	1 (2%)	1 (2%)	11 (22%)	-	1 (2%)
*M. fortuitum*	9 (18%)	-	-	1 (2%)	7 (14%)	1 (2%)	2 (4%)
*M. avium/intracellulare*	4 (8%)	2 (4%)	-	-	5 (10%)	1 (2%)	-
*M. abscessus*	3 (6%)	-	-	1 (2%)	2 (4%)	-	2 (4%)
*M. kansasii*	2 (4%)	-	-	-	2 (4%)	-	-
*M. mucogenicum*	1 (2%)	-	-	-	1 (2%)	-	-

**Legend: HIV:** Human Immunodeficiency Virus; **NA:** No Available; **a:** Genomic sequencing technique.

With regard to the treatment of NTM, evolution data were obtained for 14/50 (28%) patients, among which nine were treated with anti-tuberculosis drugs (rifampicin + isoniazid + pyrazinamide + ethambutol) and five were treated with a specific scheme for NTM, including the drugs clarithromycin, ciprofloxacin, and amikacin. A greater frequency of NTMs was observed in HIV-positive patients undergoing treatment, with the identified species being *M. fortuitum, M. gordonae, M. abscessus*, and *M. avium/intracellulare*.

### Molecular Identification of the NTMs

In this study, molecular identification tests were carried out in 22.3% (50/224) of patients. The distribution of the species identified by GenoType, PRA-*hsp*65, and genomic sequencing is shown in Table 3. The prevalent NTM species identified by biochemical tests were also confirmed using molecular techniques: *M. gordonae*, *M. fortuitum*, *Mycobacterium sp*, *M. avium/intracellulari*, *M abscessus and M. kansasii*.

**TABLE 3:** Molecular identification of nontuberculous Mycobacteria species (n = 50).

Species	Sequencing (76%)	GenoType (84%)	***PRA hsp65* (68%)**
*M. gordonae*	15 (30%)	18 (36%)	12 (24%)
*Mycobacterium sp*	12 (24%)	8 (16%)	16 (32%)
*M. fortuitum*	10 (20%)	6 (12%)	6 (12%)
*M. avium/intracellulare*	6 (12%)	8 (16%)	9 (18%)
*M. abscessus*	4 (8%)	5 (10%)	4 (8%)
*M. kansasii*	2 (4%)	2 (4%)	1 (2%)
*M. mucogenicum*	1 (2%)	1 (2%)	-
*M. scrofulaceum*	-	2 (4%)	-
*M. bohemicum*	-	-	1 (2%)
*M. nebraskense*	-	-	1 (2%)

**Legend: GenoType:** GenoType®*Mycobacterium* CM/AS; **PRA hsp65:** Polymerase Chain Reaction Restriction Analysis of the hsp65 gene.

No NTM species were identified in 32% (16/50) of the samples using the PRA-*hsp65* technique, in 16% (8/50) of samples using the GenoType method, and in 24% (12/50) of samples after genomic sequencing.

In the agreement analysis between the molecular techniques, a moderate agreement was observed between PRA*-hsp65* and GenoType (kappa = 0.65) and between GenoType and genomic sequencing (kappa = 0.60), whereas low agreement was observed between PRA *hsp65* and genomic sequencing (kappa = 0.49). The kappa´s score considered in this study was in accordance with Landis and Koch (1977),with <0.20, 0.21-0.40, 0.41-0.60, 0.61-0.80, and > 0.80 being poor, weak, moderate, good, and very good, respectively. (Table 4).

**TABLE 4:** Concordance analysis between molecular techniques.

Molecular technique	Kappa (95% CI)	Concordance
PRA *hsp65* and GenoType^a^	0.65 (0.50 to 0.80)	72.00%
GenoType and Genomic sequencing	0.60 (0.44 to 075)	68.00%
PRA *hsp65* and Genomic sequencing	0.49 (0.32 to 0.67)	60.00%

**Legend: a:** GenoType®Mycobacterium CM/AS; **CI:** Confidence Interval.

## DISCUSSION

In this study, many NTM species were isolated mainly from pulmonary clinical samples, highlighting the necessity to evaluate molecular test results with clinical information to determine potential cases of infection in patients with HIV. In addition, species implicated in pulmonary disease were also identified: *M. avium* and *M. abscessus* ^6^ ^,^ ^18^ . With regard to the potentially pathogenic NTM species, the findings of this study are similar to that of clinical practice and other studies carried out worldwide, with the most common species being *M. avium*, *M. intracellulare*, *M. kansasii*, *M. chelonae*, *M. abscessus*, *M. fortuitum*, and *M. peregrinum* ^19^ ^-^ ^21^ . 

Although nearly half of the bacterial species isolated in humans worldwide belong to MAC, the distribution of the strains, incidence, and prevalence can vary according to geographic region. In Australia, nearly 71% of the isolates belonged to MAC; where as in North America and South America, this percentage was about 52% and 31% respectively; In a recent study, in Iran, 48.4% of the isolates were *M. fortuitum*, illustrating the importance of epidemiological surveillance and disease control through techniques for the identification of NTM species ^22^ ^,^ ^23^ .

Among the NTM identified in this study, *M. gordonae* was the most frequent, both in the general population of this study and in people with HIV. This result differs from most studies conducted in Brazil, wherein the NTMs *M. kansasii* and those belonging to MAC tend to be more common ^4^ ^,^ ^20^ . 

This finding highlights the importance of using the criteria proposed by the American Thoracic Society (ATS) to differentiate colonization from contamination, as well as to conduct regional studies and monitor the true epidemiological situation of the infections caused by NTMs, especially due to the fact that such infections are often not properly reported in Brazil, with the exception of postoperative infections caused by rapid growth mycobacteria ^10^ ^,^ ^24^ .

Another important aspect of the discussion based on these results is the need to carry out molecular tests, mainly the sequencing of genes such as the *rpoB gene*, with good accuracy, for the diagnosis and epidemiological surveillance of NTM ^25^ ^,^ ^26^ .

One relevant aspect of this study was the anti-TB treatment. Risk factors associated with an increase in the probability of receiving anti-TB treatment among people with HIV were examined ^27^ . A study that evaluated the activity of new therapeutic alternatives for the treatment of NTM using d-cycloserine, clarithromycin, and combinations of both antibiotics against clinical isolates of *M. abscessus* and *M. fortuitum* highlighted the importance of accurate laboratory diagnosis and rapid methods to differentiate these mycobacteria from MAC, especially in lung samples ^23^ .

Another important aspect with respect to the frequency of NTM in patients with HIV in this study was the fact that 16% of the NTMs isolated in these patients were identified as *Mycobacterium* sp, which highlights the need to validate the rapid laboratory methods, preferably molecular methods, which can identify NTM ^8^ ^,^ ^19^ .

The variation in agreement between the results obtained by PRA-*hsp65*, GenoType, and sequencing methods illustrates the challenge for precise identification of these mycobacteria sp. as well as the importance of conducting genomic sequencing to validate the use of molecular methods ^25^ ^,^ ^26^ ^,^ ^28^ . In this context, GenoType presented a moderate agreement when compared to sequencing, which indicated that this method can be used in clinical practice to optimize the identification of NTMs ^28^ ^,^ ^29^ .

In the analysis of discordant results, the results obtained by the PRA-*hsp*65 technique differed from those obtained by genomic sequencing. In the present study, it was not possible to identify NTM species in 32% (16/50) of strains using the PRA-*hsp*65 technique. Although further studies should be conducted to confirm these results, these results indicate that the recommendations set forth by the Brazilian Ministry of Health regarding the use of the PRA-*hsp*65 technique to analyze NTMs in laboratory surveillance and routines should be reviewed. Moreover, this technique is cumbersome and costly; some NTMs can present a shared genetic profile, thus overlapping the results, and the same species can present more than one restriction profile or profiles that have not yet been described in the literature. Furthermore, because PRA-*hsp*65 is an in-house molecular test, it must be performed only by certified laboratories ^12^ . 

Nevertheless, this retrospective study had some limitations, such as limited clinical data and the difficulty in recovering a greater number of NTM isolates to conduct the genomic study, in addition to the identification of nine cases treated improperly for TB due to the incorrect identification of the NTM species. Another limitation of this study is that neither was it possible to apply the ATS clinical criteria, nor was it possible to obtain a second clinical sample to conduct the cultivation and identification of the same NTM ^10^ . 

This study identified species of MAC and *M. kansasii* species, generally considered pathogenic, in phlegm samples (Table 2). In cases of positive culture for MAC, it becomes impossible to identify the disease in patients that present a single positive culture of phlegm; however, for *M. kansasii*, a single positive culture may provide sufficient evidence to begin treatment ^30^ ^,^ ^31^ .

In conclusion, our findings highlight GenoType CM as a good method for the identification of NTM, as well as the need for the application of standardized criteria, such as those set forth by the ATS, under routine clinical and laboratory conditions to differentiate infection by NTM from colonization, as well as the need to monitor the prevalence of these mycobacterial species, especially in people with HIV, to avoid inadequate treatment and subsequent drug resistance.

## References

[1] Yamamichi T, Horio H, Asakawa A, Okui M, Harada M (2019). Clinico-bacteriological analysis for video-assisted thoracoscopic biopsy of non-tuberculous mycobacteria. J Thorac Dis.

[2] Chan ED, Iseman MD (2013). Underlying host risk factors for nontuberculous mycobacterial lung disease. Semin Respir Crit Care Med.

[3] Fusco da Costa AR, Lopes ML, Sousa MS, Suffys PN, Sales LHM, Lima KVB (2012). Pulmonary nontuberculous mycobacterial infections in the State of Para, an endemic region for tuberculosis in North of Brazil. In: AMAL, Amer. Pulmonary Infection.

[4] Fusco da Costa AR, Falkinham JO, Lopes ML, Barretto AR, Felicio JS, Sales LH (2013). Occurrence of nontuberculous mycobacterial pulmonary infection in an endemic area of tuberculosis. PLoS Negl Trop Dis.

[5] Restrepo AV, Salem JI, Ogusku MM, Gomes LF, Fraiji NA (2009). Pesquisa de Micobactérias Ambientais em água de torneira, luvas e soluções utilizadas em procedimentos cirúrgicos no Hospital Universitário Getúlio Vargas - Manaus/AM. Acta Amaz.

[6] Ministério da Saúde (MS) (2019). Boletim Epidemiológico TB-HIV 2019.

[7] Marras TK, Chedore P, Ying AM, Jamieson F (2007). Isolation prevalence of pulmonar non-tuberculous mycobacteria in Ontario, 1997-2003. Thorax.

[8] Griffith DE, Aksamit T, Brown-Elliott BA, Catanzaro A, Daley C, Gordin Fred (2007). An Official ATS/IDSA Statement: Diagnosis, Treatment, and Prevention of Nontuberculous Mycobacterial Diseases Medical Section of the American Lung Association. Am J Respir Crit Care Med.

[9] Donohue MJ, Mistry JH, Donohue JM, Connell KO, King D, Byran J (2015). Increased Frequency of Nontuberculous Mycobacteria Detection at Potable Water Taps within the United States. Environ Sci Technol.

[10] Charles LD, Jonathan MI, Christoph L, Emmanuelle C, Richard JW, Claire A (2020). Treatment of Nontuberculous Mycobacterial Pulmonary Disease: An Official ATS/ERS/ESCMID/IDSA clinical Practice Guideline. Eur Respir J.

[11] Woods GL, Brown-Elliott BA, Conville PS (2011). Teste de Susceptibilidade de Micobactérias, Nocardiae e Outros Actinomicetos Aeróbicos.

[12] Ministério da Saúde (MS), Secretaria de Vigilância em Saúde (2011). Manual de recomendações para o controle da tuberculose no Brasil.

[13] van Soolingen D, Hermans PW, de Haas PE, Soll DR, van Embden JD (1991). Occurrence and stability of insertion sequences in Mycobacterium tuberculosis complex strains: evaluation of an insertion sequence-dependent DNA polymorphism as a tool in the epidemiology of tuberculosis. J Clin Microbiol.

[14] Hain Lifesciences (2015). GenoType® Mycobacterium CM-AS Manual.

[15] Selvaraju SB, Khan IU, Yadav JS (2005). A new method for species identification and differentiation of Mycobacterium chelonae complex based on amplified hsp65 restriction analysis (AHSPRA). Mol Cell Probes.

[16] Adékambi T, Colson P, Drancourt M (2003). rpoB-based identification of nonpigmented and late-pigmenting rapidly growing mycobacteria. J Clin Microbiol.

[17] Applied Biosystems (2006). BigDye®XTerminator™ Purification Kit.

[18] Gopalaswamy R, Shanmugam S, Mondal R, Subbian S (2020). Of tuberculosis and non-tuberculous mycobacterial infections - a comparative analysis of epidemiology, diagnosis and treatment. J Biomed Sci.

[19] Puga FG, Pocente RHC, Chimara E, Bollela VR (2018). HIV-negative pulmonary disease caused by nontuberculous mycobacteria in Southern Brazil: clinical and microbiological characterization. J Thorac Dis.

[20] Ueki SY, Martins MC, Telles MA, Virgilio MC, Giampaglia CMS, Chimara E (2005). Nontuberculous mycobacteria: species diversity in São Paulo state, Brazil. J Bras Patol Med Lab.

[21] Pedro Hda S, Pereira MI, Goloni MRA, Ueki SYM, Chimara E (2008). Nontuberculous mycobacteria isolated in São José do Rio Preto, Brazil between 1996 and 2005. J Bras Pneumol.

[22] Hoefsloot W, van Ingen J, Andrejak C, Angeby K, Bauriaud R, Bemeret P (2013). The geographic diversity of nontuberculous mycobacteria isolated from pulmonary samples: an NTM-NET collaborative study. Eur Respir J.

[2] Khosravi AD, Mirsaeidi M, Farahani A, Tabandeh MR, Mohajeri P, Saeed Shoja S (2018). Prevalence of nontuberculous mycobacteria and high efficacy of D-cycloserine and its synergistic effect with clarithromycin against Mycobacterium fortuitum and Mycobacterium abscessus. Infect Drug Resist.

[24] ANVISA. Agência Nacional de Vigilância Sanitária (2009). Infecções por Micobactérias de Crescimento Rápido: Fluxo de Notificações, Diagnósticos Clínico, Microbiológicos e Tratamento.

[25] Dastranj M, Farahani A, Shahraki AH, Atashi S, Mohajeri P (2018). Molecular identification and distribution of non-tuberculous mycobacteria isolated from clinical specimens by PCR-sequencing method in West of Iran. Clin Respir J.

[26] Mohajeri P, Yazdani L, Shahraki AH, Alvandi A, Atashi S, Farahani A (2017). Verification of frequency in species of nontuberculous mycobacteria in Kermanshah drinking water supplies using the PCR-sequencing method. Microb Drug Resist.

[27] Agizew T, Boyd R, Mathebula U, Mathoma A, Basotli J, Serumola C (2020). Outcomes of HIV-positive patients with non-tuberculous mycobacteria positive culture who received anti-tuberculous treatment in Botswana: Implications of using diagnostic algorithms without non-tuberculous mycobacteria. PLoS One.

[28] Yang M, Huh HJ, Kwon HJ, Kim J, Song DJ, Koh W (2016). Comparative evaluation of the AdvanSure Mycobacteria GenoBlot assay and the GenoType Mycobacterium CM/AS assay for the identification of non-tuberculous mycobacteria. J Med Microbiol.

[29] Lee AS, Jelfs P, Sintchenko V, Gilbert GL (2009). Identification of non-tuberculous mycobacteria: utility of the GenoType Mycobacterium CM/AS assay compared with HPLC and 16S rRNA gene sequencing. J Med Microbiol.

[30] Jankovic M, Sabol I, Zmak L, Jankovic VK, Jakopovic M, Obrovac M (2016). Microbiological criteria in non-tuberculous mycobacteria pulmonary disease: a tool for diagnosis and epidemiolog. Int J Tuberc Lung Dis.

[31] Lee SR, Yang CY, Shu CC, Lin CK, Wen YF, Lee SW (2015). Factors associated with subsequent nontuberculous mycobacterial lung disease in patients with a single sputum isolate on initial examination. Clin Microbiol Infect.

