# Supplementary material for: Molecular characterization of nontuberculous Mycobacteria in a tuberculosis and HIV reference unit in the State of Amazonas, Brazil
Source: Rev Soc Bras Med Trop. 2022 Aug 5;55:e0613-2021. doi: 10.1590/0037-8682-0613-2021 (PMC9344947; doi:10.1590/0037-8682-0613-2021)
Supplement: Supplementary file 1 [file 1678-9849-rsbmt-55-e0613-2021-supp1.pdf]

**SUPPLEMENTARY MATERIAL 1:** Division of *Mycobacterium tuberculosis* complex species of nontuberculous mycobacteria.

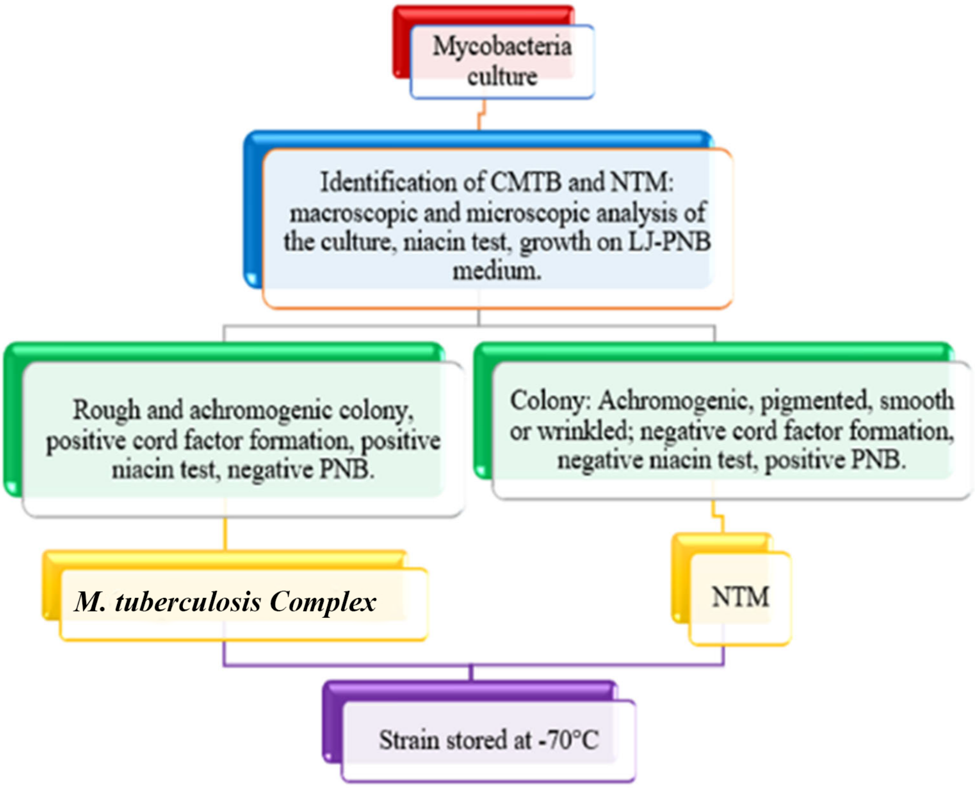


PNB: Löwenstein-Jensen medium with o p-nitrobenzoic acid; CMTB: *Mycobacterium tuberculosis* complex; NTM: NonTuberculous mycobacteria

Adapted: Brazil. National Manual for Laboratory Surveillance of Tuberculosis and Other Mycobacteria - Ministry of Health, 2008.

**SUPPLEMENTARY MATERIAL 2:** Gel patterns of polymerase chain reaction restriction analysis of the *hsp65* gene- *PRA-hsp65.*

**Ladder 1H 1B 2H 2B 3H 3B 4H 4B 5H 5B 6H 6B 7H 7B 8H 8B 9H 9B Ladder**

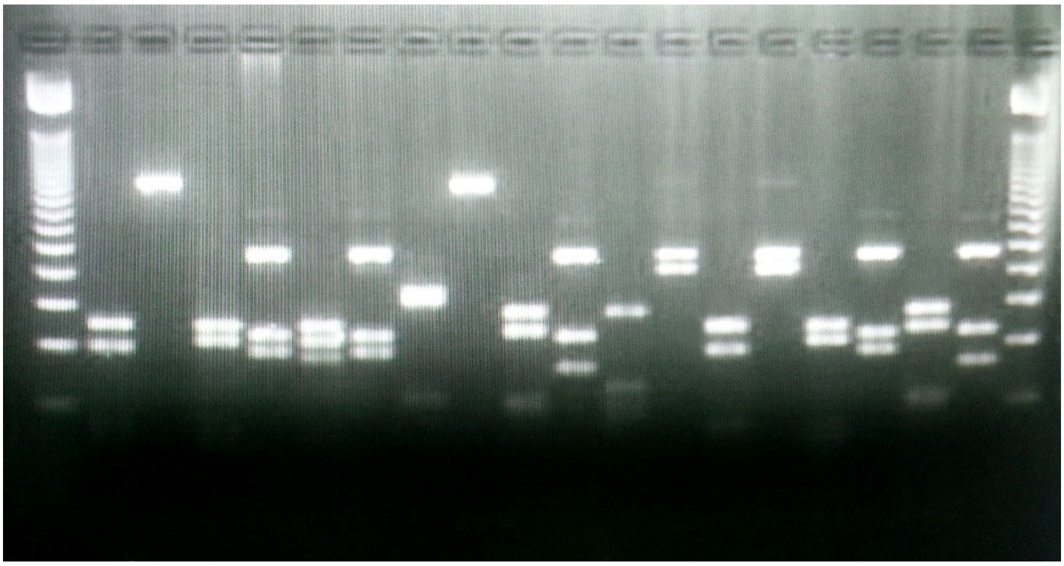

Ladder:50 bp

| | **Band Pattern (BP)** | | **Most likely microbial agents - PRA SITE** |
|---|---|---|---|
| | *BsteII* | *HaeIII* | |
| **1** | 440 | 125/100 | *Inconclusive* |
| **2** | 235/120/100 | 130/115 | *M. gordonae* |
| **3** | 235/120/100 | 130/110/95 | *M. gordonae* |
| **4** | 440 | 160/60 | *Inconclusive* |
| **5** | 235/120/85 | 140/120/60 | *M. fortuituim* |
| **6** | 235/210 | 145/70/60/55 | *M. abscessus* |
| **7** | 235/210 | 130/105 | *M. avium* |
| **8** | 235/120/100 | 130/115 | *M. gordonae* |
| **9** | 235/120/85 | 140/120/60 | *M. fortuituim* |

Ladder 10H 10B 11H 11B 12H 12B 13H 13B 14H 14B 15H 15B 16H 16B 17H 17B 18H 18B Ladder

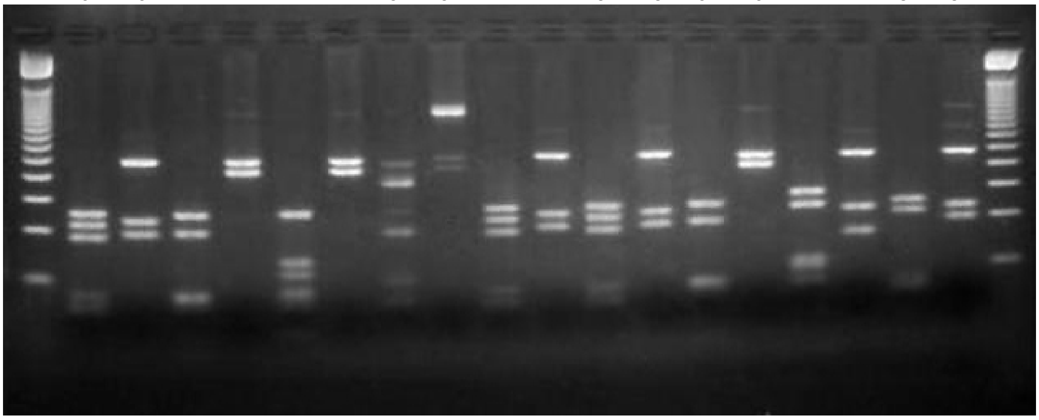

**Repetition and more run time to confirm some band patterns:**

Ladder 10H 10B 11H 11B 12H 12B 13H 13B 14H 14B 15H 15B 16H 16B 17H 17B 18H 18B Ladder

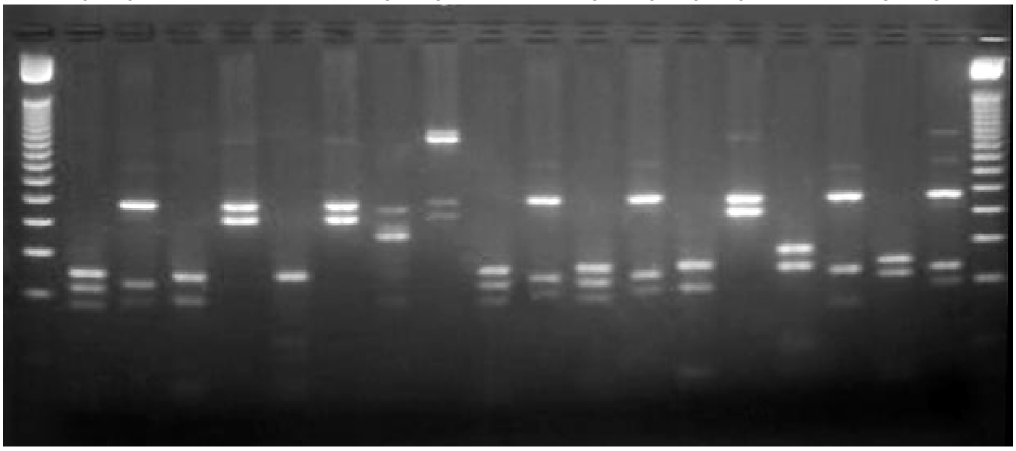

Ladder: 50 bp

| | **Band Pattern (BP)** | | **Most Likely Microbial Agents - PRA SITE** |
|---|---|---|---|
| | *BsteII* | *HaeIII* | |
| **10** | 235/120/100 | 130/110/95 | *M. gordonae* |
| **11** | 235/210 | 145/105 | *M. bohemicum* |
| **12** | 235/210 | 145/70/60/55 | *M. abscessus* |
| **13** | No lecture | 245/200/130/100 | Inconclusivo |
| **14** | 235/120/100 | 130/110/95 | *M. gordonae* |
| **15** | 235/120/100 | 130/110/95 | *M. gordonae* |
| **16** | 235/210 | 130/105 | *M. avium* |

| | | | |
|---|---|---|---|
| **17** | 235/120/85 | 140/125/60 | *M. fortuitum* |
| **18** | 235/120/100 | 130/110/95 | *M. gordonae* |

Ladder 37H 37B 38H 38B 39H 39B 40H 40B 41H 41B 42H 42B 43H 43B 44H 44B 45H 45B Ladder

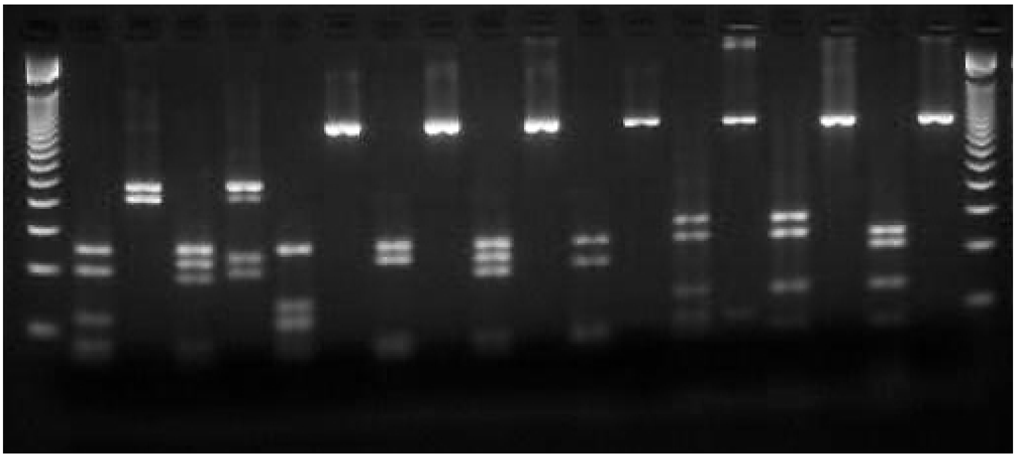

Ladder 46H 46B 47H 47B 48H 48B 49H 49B 50H 50B 51H 51B 52H 52B 53H 53B54H 54B Ladder

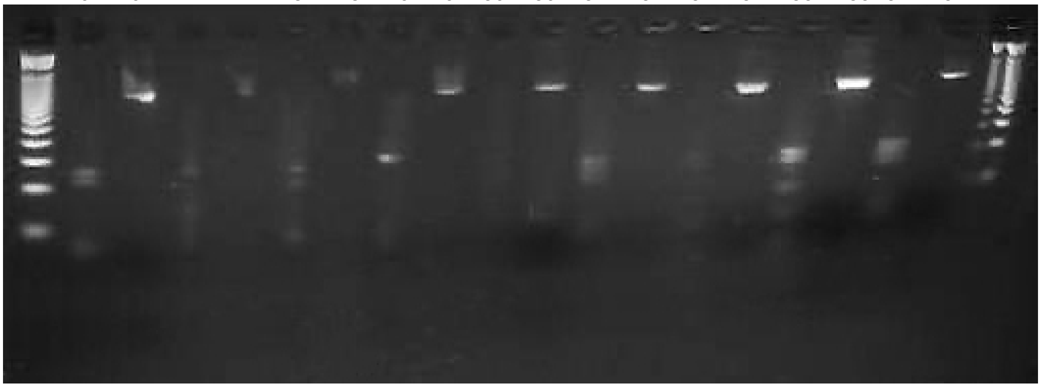

Hard-to-see, repeated gels.

Ladder 55H 55B 56H 56B Ladder

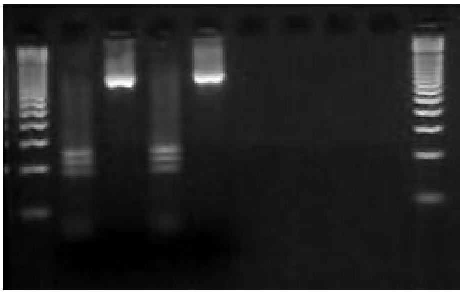

**Ladder: 50 bp**

**Repetitions:**

**(Electrophoresis for samples 46 to 56 was repeated with 50 and 25 bp marker. In the gels, the first ladder is 50 bp and the second is 25 bp.)**

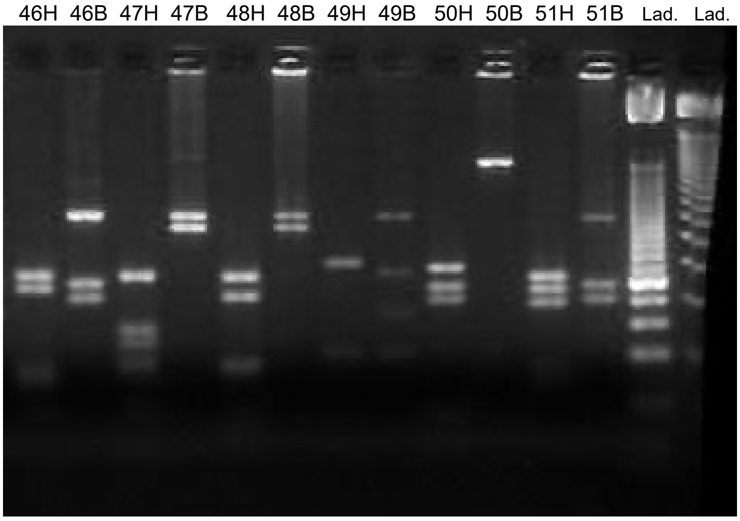


**After increasing the run time.**

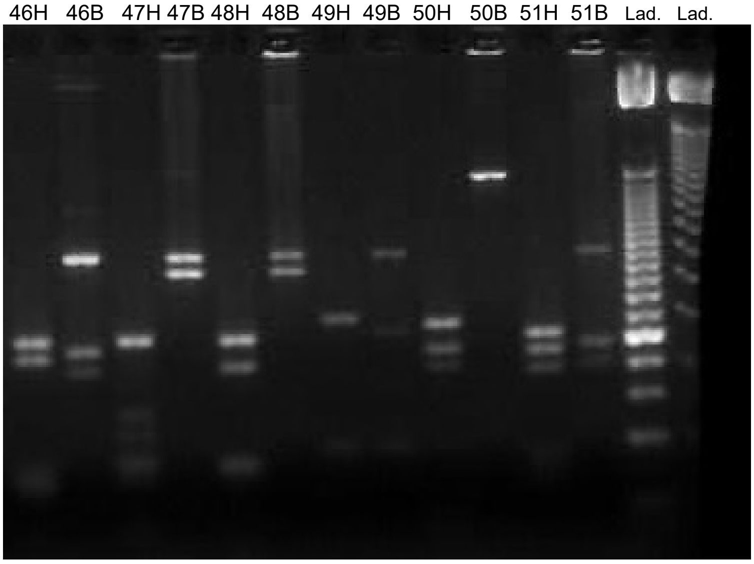

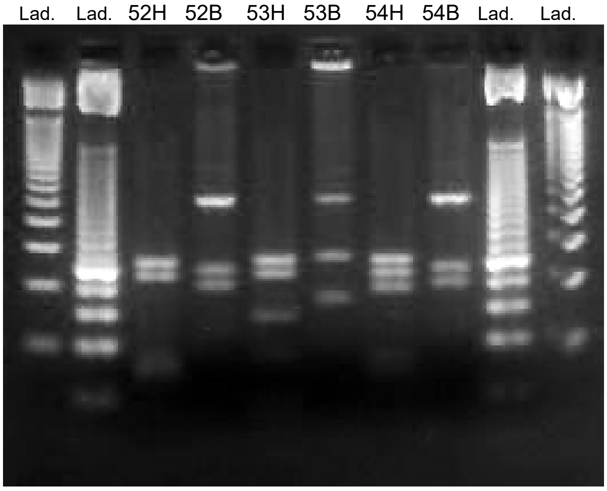


**After increasing the run time.**

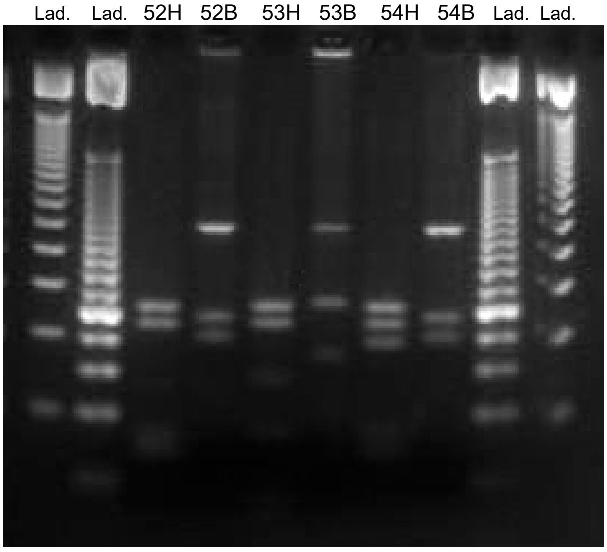


| | Band Pattern (BP) | | Most likely microbial aagents - PRA SITE |
|---|---|---|---|
| | *BsteII* | *HaeIII* | |
| **37** | 235/210 | 130/105/60 | *M. avium* ou *M. colombiense* |
| **38** | 250/220/120/100 | 125/100/90 | Inconclusive |
| **39** | 440 | 125/60/55 | Inconclusive |
| **40** | 440 | 125/100 | Inconclusive |
| **41** | 440 | 125/100/90 | Inconclusive |
| **42** | 440 | 125/90 | Inconclusive |
| **43** | 440 | 150/120/60 | Inconclusive |

| | | | |
|---|---|---|---|
| **44** | 440 | 150/120/60 | Inconclusive |
| **45** | 440 | 125/105/60 | *M. nebraskense* |
| **46** | 235/120/100 | 130/115 | *M. gordonae* |
| **47** | 250/230 | 125/60/45 | Inconclusive |
| **48** | 235/210 | 125/105 | *M. avium* |
| **49** | 250/150/50 | 155/50 | Inconclusive |
| **50** | 440 | 150/125/120 | Inconclusive |
| **51** | 235/120/100 | 130/110/95 | *M. gordonae* |
| **52** | 235/120/100 | 130/115 | *M. gordonae* |
| **53** | 235/130/85 | 130/105/70 | *M. kansasii* |
| **54** | 235/120/100 | 130/110/95 | *M. gordonae* |
| **55** | 440 | 125/100/90 | Inconclusive |
| **56** | 440 | 125/100/90 | Inconclusive |

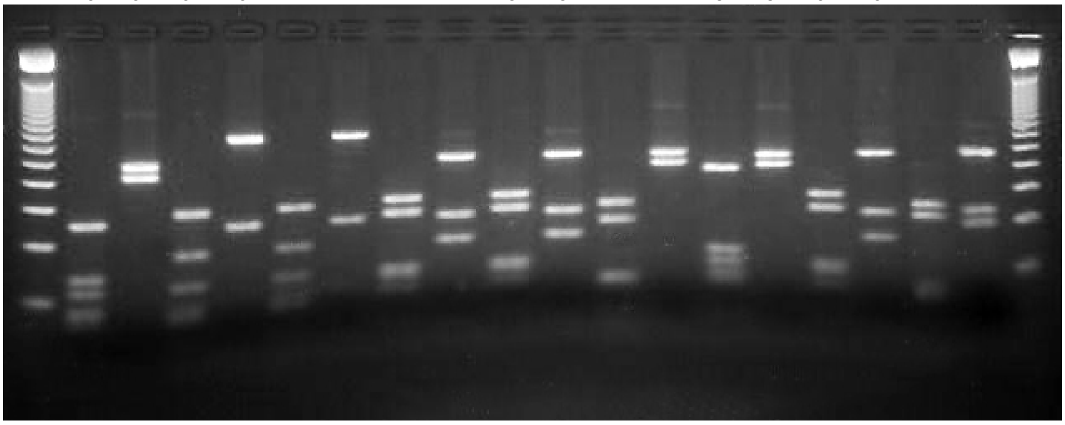


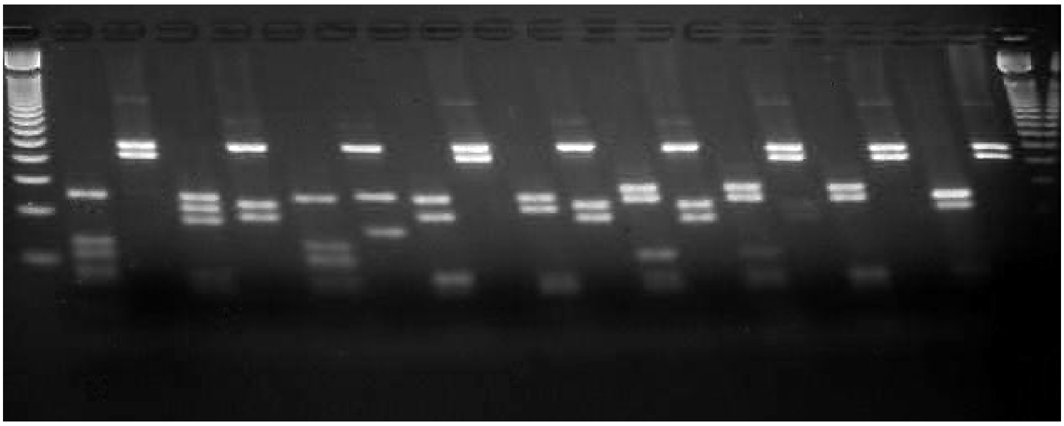


| | Band Pattern (BP) | | Most likely microbial agents - PRA SITE |
|---|---|---|---|
| | *BsteII* | *HaeIII* | |
| **19** | 235/210 | 145/70/60/55 | *M. abscessus* |
| **20** | 320/130 | 140/90/60/45 | Inconclusive |
| **21** | 320/130 | 140/90/60/45 | Inconclusive |
| **22** | 235/120/85 | 145/120/60 | *M. fortuituim* |
| **23** | 235/120/85 | 145/120/60 | *M. fortuituim* |
| **24** | 235/210 | 130/105 | *M. avium* |
| **25** | 235/210 | 200/70/60/50 | *M. abscessus* |
| **26** | 235/120/85 | 145/120/60 | *M. fortuitum* |
| **27** | 235/120/100 | 130/110 | *M. gordonae* |

| | | | |
|---|---|---|---|
| **28** | 235/210 | 145/70/60/55 | *M. abscessus* |
| **29** | 235/120/100 | 130/110/95 | *M. gordonae* |
| **30** | 235/130/85 | 130/70/60 | Inconclusiva |
| **31** | 235/210 | 130/105/60 | *M. avium* |
| **32** | 235/120/100 | 130/115 | *M. gordonae* |
| **33** | 250/110/90 | 135/100 | Inconclusive |
| **34** | 235/210 | 145/130 | *M. saskatchewanense, M. seoulense, M. simiae, M. intracellulare, M. interjectum, M. intermedium ou M. avium* |
| **35** | 235/210 | 145/130 | *M. saskatchewanense, M. seoulense, M. simiae, M. intracellulare, M. interjectum, M. intermedium ou M. avium* |
| **36** | 235/210 | 145/130 | *M. saskatchewanense, M. seoulense, M. simiae, M. intracellulare, M. interjectum, M. intermedium ou M. avium* |
