# Supplementary material for: Molecular characterization of nontuberculous Mycobacteria in a tuberculosis and HIV reference unit in the State of Amazonas, Brazil
Source: Rev Soc Bras Med Trop. 2022 Aug 5;55:e0613-2021. doi: 10.1590/0037-8682-0613-2021 (PMC9344947; doi:10.1590/0037-8682-0613-2021)
Supplement: Supplementary file 2 [file 1678-9849-rsbmt-55-e0613-2021-supp2.pdf]

SUPPLEMENTARY MATERIAL 2: Gel patterns of polymerase chain reaction restriction analysis of the *hsp65* gene- *PRA-hsp65.*

Ladder 1H 1B 2H 2B 3H 3B 4H 4B 5H 5B 6H 6B 7H 7B 8H 8B 9H 9B Ladder

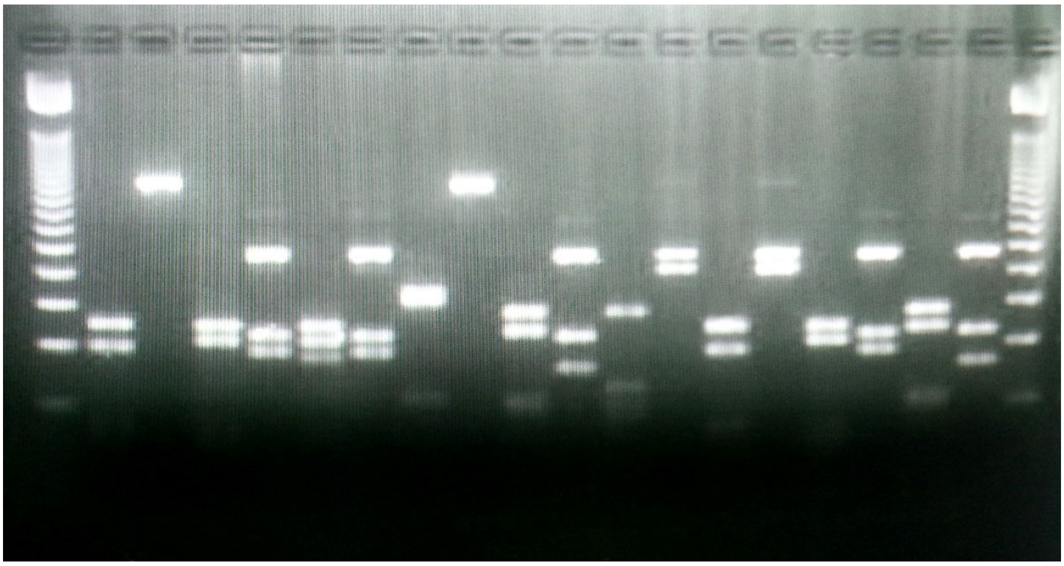

Ladder:50 bp

	Band Pattern (BP)		Most likely microbial agents - PRA SITE
	BsteII	*HaeIII*	
**1**	440	125/100	*Inconclusive*
**2**	235/120/100	130/115	*M. gordonae*
**3**	235/120/100	130/110/95	*M. gordonae*
**4**	440	160/60	*Inconclusive*
**5**	235/120/85	140/120/60	*M. fortuituim*
**6**	235/210	145/70/60/55	*M. abscessus*
**7**	235/210	130/105	*M. avium*
**8**	235/120/100	130/115	*M. gordonae*
**9**	235/120/85	140/120/60	*M. fortuituim*

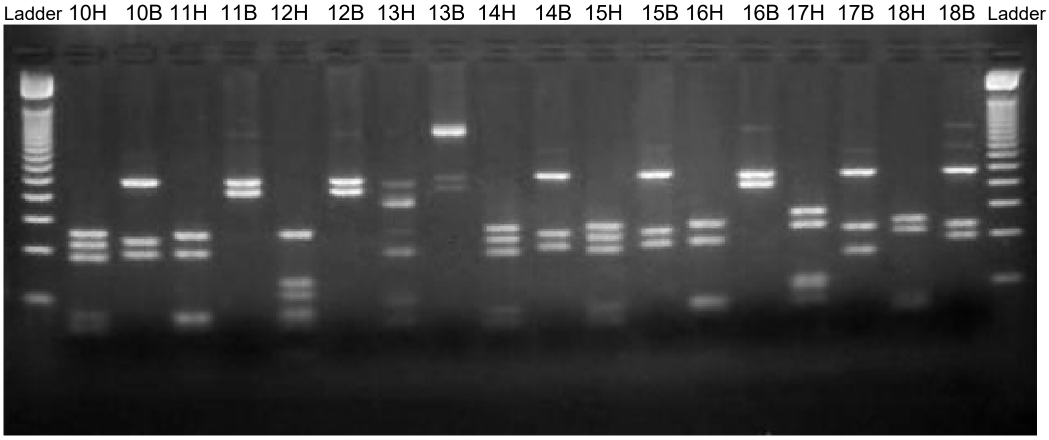

Repetition and more run time to confirm some band patterns:

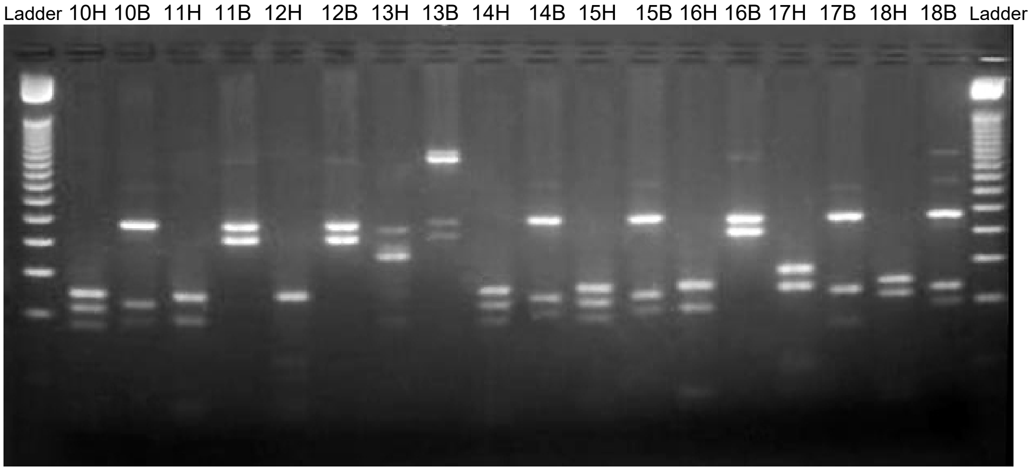

Ladder: 50 bp

	Band Pattern (BP)		Most Likely Microbial Agents - PRA SITE
	BsteII	*HaeIII*	
**10**	235/120/100	130/110/95	*M. gordonae*
**11**	235/210	145/105	*M. bohemicum*
**12**	235/210	145/70/60/55	*M. abscessus*
**13**	No lecture	245/200/130/100	Inconclusivo
**14**	235/120/100	130/110/95	*M. gordonae*
**15**	235/120/100	130/110/95	*M. gordonae*
**16**	235/210	130/105	*M. avium*

**17**	235/120/85	140/125/60	*M. fortuitum*
**18**	235/120/100	130/110/95	*M. gordonae*

Ladder 37H 37B 38H 38B 39H 39B 40H 40B 41H 41B 42H 42B 43H 43B 44H 44B 45H 45B Ladder

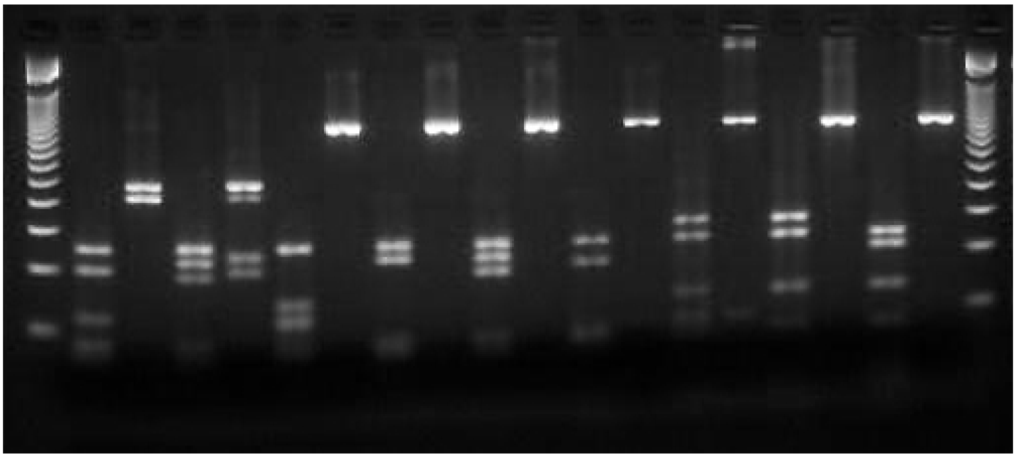

Ladder 46H 46B 47H 47B 48H 48B 49H 49B 50H 50B 51H 51B 52H 52B 53H 53B54H 54B Ladder

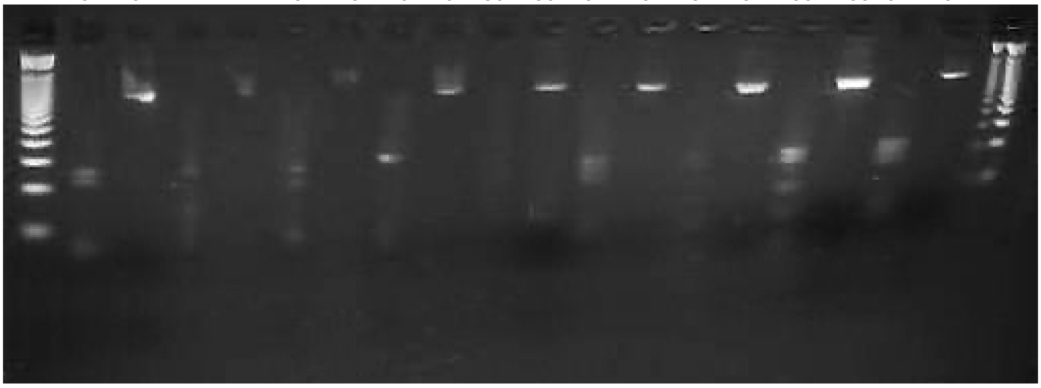

Hard-to-see, repeated gels.

Ladder 55H 55B 56H 56B Ladder

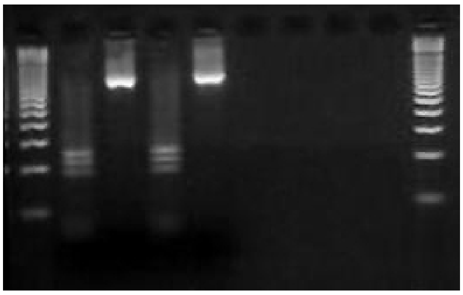

Ladder: 50 bp

Repetitions:

(Electrophoresis for samples 46 to 56 was repeated with 50 and 25 bp marker. In the gels, the first ladder is 50 bp and the second is 25 bp.)

46H 46B 47H 47B 48H 48B 49H 49B 50H 50B 51H 51B Lad. Lad.

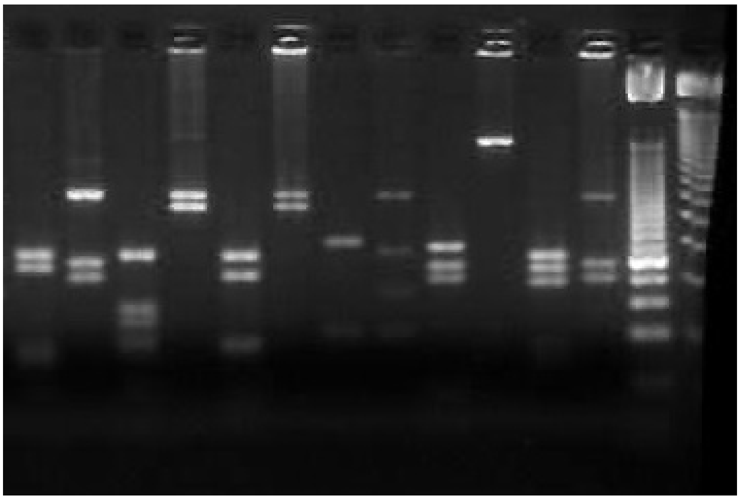

After increasing the run time.

46H 46B 47H 47B 48H 48B 49H 49B 50H 50B 51H 51B Lad. Lad.

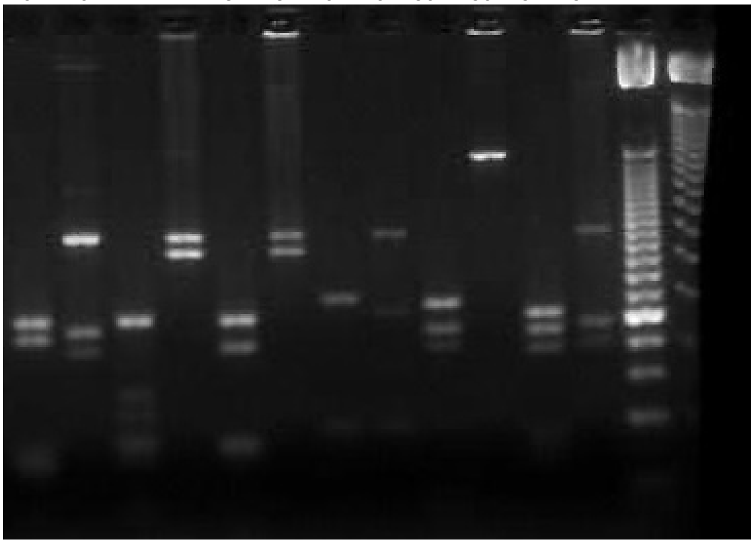

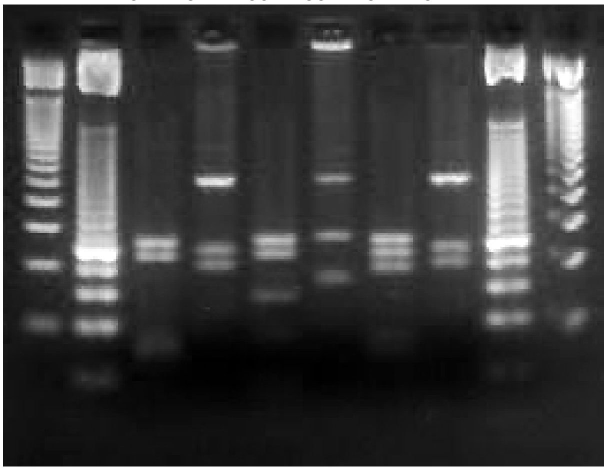

After increasing the run time.

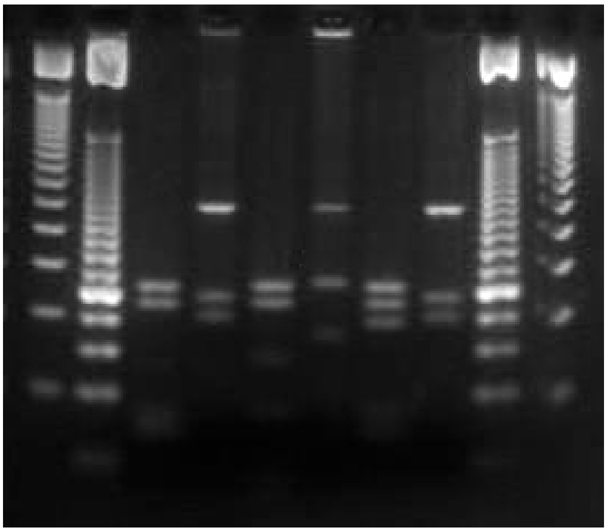

	Band Pattern (BP)		Most likely microbial aagents - PRA SITE
	BsteII	*HaeIII*	
**37**	235/210	130/105/60	*M. avium* ou *M. colombiense*
**38**	250/220/12 0/100	125/100/90	Inconclusive
**39**	440	125/60/55	Inconclusive
**40**	440	125/100	Inconclusive
**41**	440	125/100/90	Inconclusive
**42**	440	125/90	Inconclusive
**43**	440	150/120/60	Inconclusive

**44**	440	150/120/60	Inconclusive
**45**	440	125/105/60	*M. nebraskense*
**46**	235/120/100	130/115	*M. gordonae*
**47**	250/230	125/60/45	Inconclusive
**48**	235/210	125/105	*M. avium*
**49**	250/150/50	155/50	Inconclusive
**50**	440	150/125/120	Inconclusive
**51**	235/120/100	130/110/95	*M. gordonae*
**52**	235/120/100	130/115	*M. gordonae*
**53**	235/130/85	130/105/70	*M. kansasii*
**54**	235/120/100	130/110/95	*M. gordonae*
**55**	440	125/100/90	Inconclusive
**56**	440	125/100/90	Inconclusive

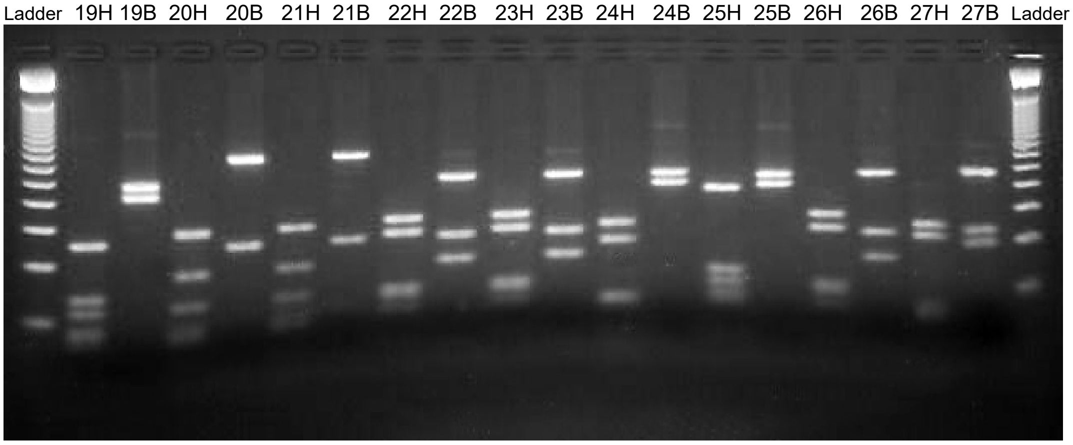

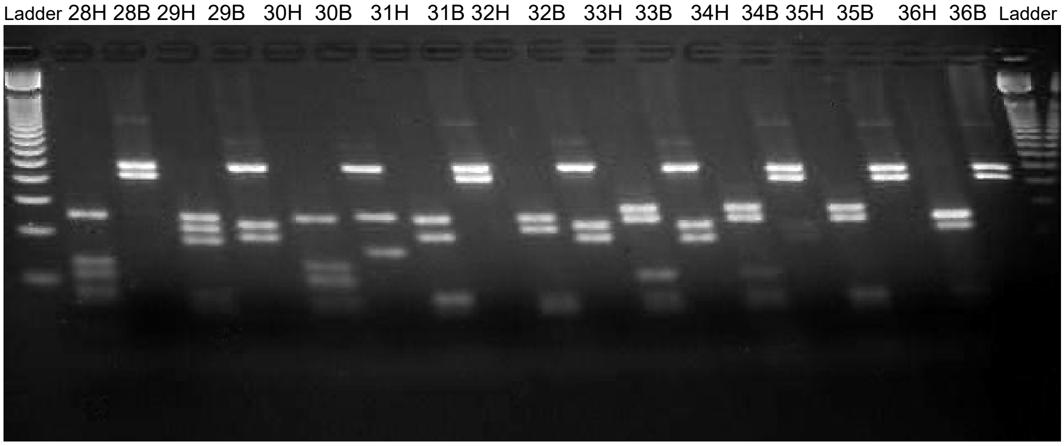

	Band Pattern (BP)		Most likely microbial agents - PRA SITE
	BsteII	*HaeIII*	
**19**	235/210	145/70/60/55	*M. abscessus*
**20**	320/130	140/90/60/45	Inconclusive
**21**	320/130	140/90/60/45	Inconclusive
**22**	235/120/85	145/120/60	*M. fortuituim*
**23**	235/120/85	145/120/60	*M. fortuituim*
**24**	235/210	130/105	*M. avium*
**25**	235/210	200/70/60/50	*M. abscessus*
**26**	235/120/85	145/120/60	*M. fortuitum*
**27**	235/120/100	130/110	*M. gordonae*

**28**	235/210	145/70/60/55	*M. abscessus*
**29**	235/120/100	130/110/95	*M. gordonae*
**30**	235/130/85	130/70/60	Inconclusiva
**31**	235/210	130/105/60	*M. avium*
**32**	235/120/100	130/115	*M. gordonae*
**33**	250/110/90	135/100	Inconclusive
**34**	235/210	145/130	*M. saskatchewanense, M. seoulense, M. simiae, M. intracellulare, M. interjectum, M. intermedium ou M. avium*
**35**	235/210	145/130	*M. saskatchewanense, M. seoulense, M. simiae, M. intracellulare, M. interjectum, M. intermedium ou M. avium*
**36**	235/210	145/130	*M. saskatchewanense, M. seoulense, M. simiae, M. intracellulare, M. interjectum, M. intermedium ou M. avium*
